# Supplementary material for: Mitochondrial DNA Changes in Genes of Respiratory Complexes III, IV and V Could Be Related to Brain Tumours in Humans
Source: Int J Mol Sci. 2022 Oct 12;23(20):12131. doi: 10.3390/ijms232012131 (PMC9603055; doi:10.3390/ijms232012131)
Supplement: Supplementary file 1 [file ijms-23-12131-s001.zip › Figure S1.pdf]

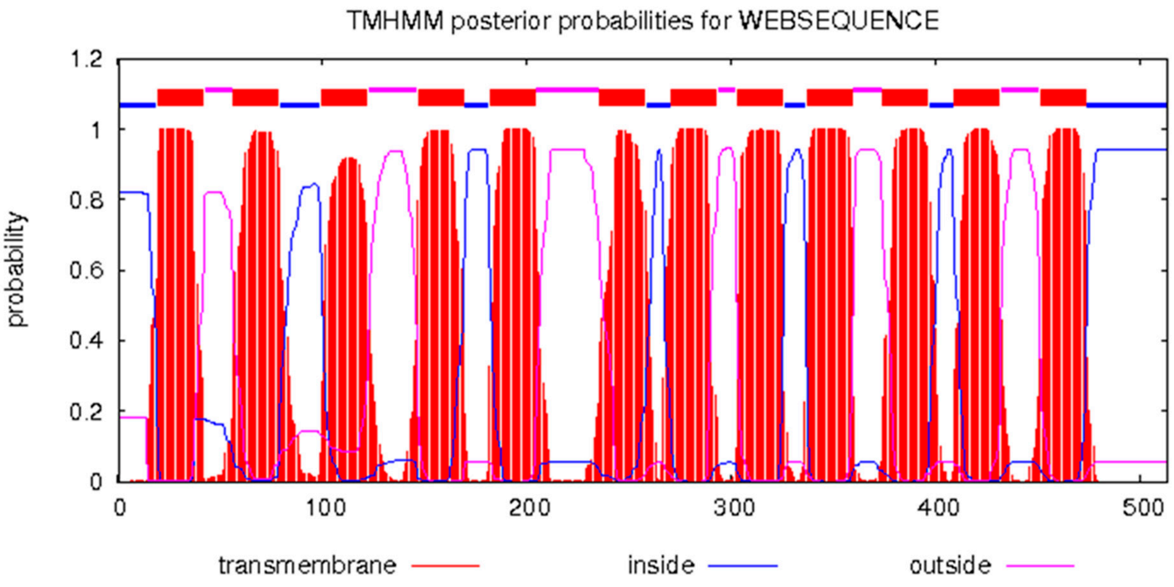


```
# WEBSEQUENCE Length: 513
# WEBSEQUENCE Number of predicted TMHs:  12
# WEBSEQUENCE Exp number of AAs in TMHs: 269.49928
# WEBSEQUENCE Exp number, first 60 AAs:  25.21909
# WEBSEQUENCE Total prob of N-in:        0.82046
# WEBSEQUENCE POSSIBLE N-term signal sequence
WEBSEQUENCE     TMHMM2.0        inside       1    19
WEBSEQUENCE     TMHMM2.0        TMhelix     20    42
WEBSEQUENCE     TMHMM2.0        outside     43    56
WEBSEQUENCE     TMHMM2.0        TMhelix     57    79
WEBSEQUENCE     TMHMM2.0        inside      80    99
WEBSEQUENCE     TMHMM2.0        TMhelix    100   122
WEBSEQUENCE     TMHMM2.0        outside    123   146
WEBSEQUENCE     TMHMM2.0        TMhelix    147   169
WEBSEQUENCE     TMHMM2.0        inside     170   181
WEBSEQUENCE     TMHMM2.0        TMhelix    182   204
WEBSEQUENCE     TMHMM2.0        outside    205   235
WEBSEQUENCE     TMHMM2.0        TMhelix    236   258
WEBSEQUENCE     TMHMM2.0        inside     259   270
WEBSEQUENCE     TMHMM2.0        TMhelix    271   293
WEBSEQUENCE     TMHMM2.0        outside    294   302
WEBSEQUENCE     TMHMM2.0        TMhelix    303   325
WEBSEQUENCE     TMHMM2.0        inside     326   336
WEBSEQUENCE     TMHMM2.0        TMhelix    337   359
WEBSEQUENCE     TMHMM2.0        outside    360   373
WEBSEQUENCE     TMHMM2.0        TMhelix    374   396
WEBSEQUENCE     TMHMM2.0        inside     397   408
WEBSEQUENCE     TMHMM2.0        TMhelix    409   431
WEBSEQUENCE     TMHMM2.0        outside    432   450
WEBSEQUENCE     TMHMM2.0        TMhelix    451   473
WEBSEQUENCE     TMHMM2.0        inside     474   513
```

Figure S1. Reference protein CO1 according to the Cambridge sequence (red font indicates the places where the amino acid shift took place).
