# Supplementary material for: Mitochondrial DNA Changes in Genes of Respiratory Complexes III, IV and V Could Be Related to Brain Tumours in Humans
Source: Int J Mol Sci. 2022 Oct 12;23(20):12131. doi: 10.3390/ijms232012131 (PMC9603055; doi:10.3390/ijms232012131)
Supplement: Supplementary file 1 [file ijms-23-12131-s001.zip › Figure S2.pdf]

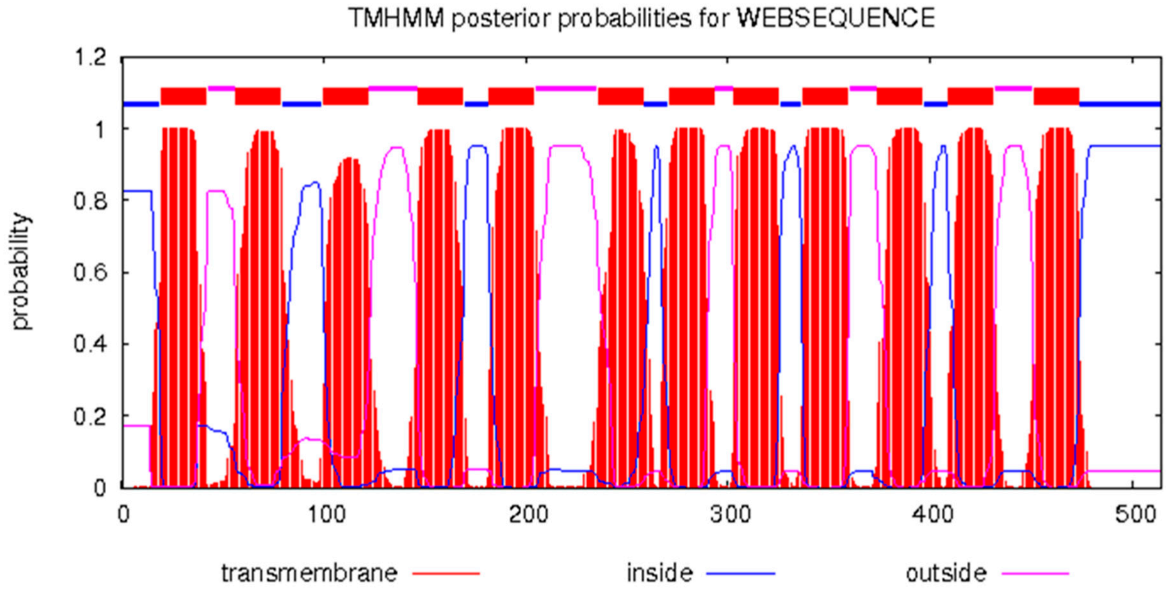


```
# WEBSEQUENCE Length: 514
# WEBSEQUENCE Number of predicted TMHs:  12
# WEBSEQUENCE Exp number of AAs in TMHs: 269.49591
# WEBSEQUENCE Exp number, first 60 AAs:  25.20909
# WEBSEQUENCE Total prob of N-in:        0.82688
# WEBSEQUENCE POSSIBLE N-term signal sequence
```

WEBSEQUENCE	TMHMM2.0	inside	1	19
WEBSEQUENCE	TMHMM2.0	TMhelix	20	42
WEBSEQUENCE	TMHMM2.0	outside	43	56
WEBSEQUENCE	TMHMM2.0	TMhelix	57	79
WEBSEQUENCE	TMHMM2.0	inside	80	99
WEBSEQUENCE	TMHMM2.0	TMhelix	100	122
WEBSEQUENCE	TMHMM2.0	outside	123	146
WEBSEQUENCE	TMHMM2.0	TMhelix	147	169
WEBSEQUENCE	TMHMM2.0	inside	170	181
WEBSEQUENCE	TMHMM2.0	TMhelix	182	204
WEBSEQUENCE	TMHMM2.0	outside	205	235
WEBSEQUENCE	TMHMM2.0	TMhelix	236	258
WEBSEQUENCE	TMHMM2.0	inside	259	270
WEBSEQUENCE	TMHMM2.0	TMhelix	271	293
WEBSEQUENCE	TMHMM2.0	outside	294	302
WEBSEQUENCE	TMHMM2.0	TMhelix	303	325
WEBSEQUENCE	TMHMM2.0	inside	326	336
WEBSEQUENCE	TMHMM2.0	TMhelix	337	359
WEBSEQUENCE	TMHMM2.0	outside	360	373
WEBSEQUENCE	TMHMM2.0	TMhelix	374	396
WEBSEQUENCE	TMHMM2.0	inside	397	408
WEBSEQUENCE	TMHMM2.0	TMhelix	409	431
WEBSEQUENCE	TMHMM2.0	outside	432	450
WEBSEQUENCE	TMHMM2.0	TMhelix	451	473
WEBSEQUENCE	TMHMM2.0	inside	474	514

Figure S2. The protein CO1 with polymorphism STP514K (red font indicates the shift of amino acids in the protein).
