# Supplementary material for: Mitochondrial DNA Changes in Genes of Respiratory Complexes III, IV and V Could Be Related to Brain Tumours in Humans
Source: Int J Mol Sci. 2022 Oct 12;23(20):12131. doi: 10.3390/ijms232012131 (PMC9603055; doi:10.3390/ijms232012131)
Supplement: Supplementary file 1 [file ijms-23-12131-s001.zip › Table S1.pdf]

Table S1. Summary of all detected changes in the cytochrome b gene, cytochrome oxidase complex genes and the *ATP6* gene. Polimorphisms are written in normal font. Mutations are marked in italic**.** Missense changes are marked in bold. Heteroplasmia: tumor:(1) G-72,8%, A-27,2%; sample of blood: G-45%, A- 55% .(2) T – 72.5%, C – 27.5%; (3) tumor: A – 59.5%, G – 40.5%. (4) : A -64,5%, G – 35,5%;

**Change in the gene**	**The number of patients with diagnosed polymorphism with the diagnosis of tumor grade**			**The number of all patients in whom polymorphism was detected**	**Region**	**Amino acid change**	**Type of change**	**Described in the available literature**
	**II**	**III**	**IV**					
**T14766C**	**4/7**	**4/6**	**3/17**	**11 /30**	***MT-CYB***	**I —7— T**	**missense**	**yes**
**T14798C**	**1/7**	**1/6**	**0/17**	**2/30**	***MT-CYB***	**F —18— L**	**missense**	**yes**
A14893G	0/7	0/6	1/17	1/30	*MT-CYB*	L —49— L	synonymous	yes
T14971C	0/7	0/6	1/17	1/30	*MT-CYB*	Y —75— Y	synonymous	yes
**A14793G**	**1/7**	**0/6**	**2/17**	**3/30**	***MT-CYB***	**H —16— R**	**missense**	**yes**
G14905A	1/7	1/6	0/17	2/30	*MT-CYB*	M —53— M	synonymous	yes
A15052G	0/7	1/6	0/17	1/30	*MT-CYB*	L —102— L	synonymous	yes
G15106A	0/7	1/6	0/17	1/30	*MT-CYB*	L —120— L	synonymous	yes
**A15218G**	**1/7**	**0/6**	**2/17**	**3/30**	***MT-CYB***	**T —158— A**	**missense**	**yes**
**A15326G**	**7/7**	**6/6**	**17/17**	**30 /30**	***MT-CYB***	**T —194— A**	**missense**	**yes**
A15244G	0/7	0/6	1/17 (1)	1/30	*MT-CYB*	G —166— G	synonymous	yes
G15355A	0/7	0/6	1/17	1/30	*MT-CYB*	T —203— T	synonymous	yes
**C15452A**	**1/7**	**0/6**	**2/17**	**3/30**	***MT-CYB***	**L —236— I**	**missense**	**yes**
**C15459T**	**0/7**	**0/6**	**1/17**	**1/30**	***MT-CYB***	**S —238— F**	**missense**	**yes**
C15499T	0/7	1/6	0/17	1/30	*MT-CYB*	G —251— G	synonymous	not
A15607G	1/7	1/6	0/17	2/30	*MT-CYB*	K —287— K	synonymous	yes
**A15656G**	**0/7**	**0/6**	**1/17**	**1/30**	***MT-CYB***	**I —304— V**	**missense**	**not**
T15663C	***0/7***	***0/6***	***1/17 (2)***	***1/30***	***MT-CYB***	***I —306— T***	***missense***	***no***
**A15758G**	**0/7**	**0/6**	**1/17**	**1/30**	***MT-CYB***	**I —338— V**	**missense**	**yes**
C15833T	0/7	0/6	1/17	1/30	*MT-CYB*	L —363— L	synonymous	yes
**Sum of detected changes in *MT-CYB***	17	16	35	67				
T5999C	1/7	0/6	1/17	2/30	*MT-CO1*	A —32— A	synonymous	yes
A6047G	1/7	0/6	1/17	2/30	*MT-CO1*	L —48— L	synonymous	yes
C6296T	0/7	0/6	1/17	1/30	*MT-CO1*	P —131— P	synonymous	yes

A6359G	0/7	1/6	0/17	1/30	*MT-CO1*	L –152– L	synonymous	yes
T6392C	0/7	1/6	0/17	1/30	*MT-CO1*	N –163– N	synonymous	yes
C6455T	0/7	1/6	0/17	1/30	*MT-CO1*	F –184– F	synonymous	yes
A6461G	0/7	0/6	1/17	1/30	*MT-CO1*	W –186– W	synonymous	not
T6620C	0/7	0/6	1/17	1/30	*MT-CO1*	G –239– G	synonymous	yes
G6755A	*0/7*	*0/6*	*1/17 (3)*	*1/30*	*MT-CO1*	*G –284– G*	*synonymous*	*yes*
T6776C	0/7	0/6	1/17	1/30	*MT-CO1*	H –291– H	synonymous	yes
C7028T	4/7	5/6	4/17	13/30	*MT-CO1*	A –375– A	synonymous	yes
**G7075C**	**0/6**	**0/6**	**1/17**	**1/30**	***MT-CO1***	**G –391– A**	**missense**	**not**
A7055G	0/7	1/6	0/17	1/30	*MT-CO1*	G –384– G	synonymous	yes
**G7444A**	**0/7**	**0/6**	**1/17**	**1/30**	***MT-CO1***	**STP –514– K**	**missense**	**yes**
T8038G	0/7	1/6	0/17	1/30	*MT-CO2*	R –150– R	synonymous	not
G8251A	1/7	0/6	0/17	1/30	*MT-CO2*	W –222– W	synonymous	yes
G8269A	0/7	0/6	1/17	1/30	*MT-CO2*	STP –228– STP	synonymous	yes
G9329A	0/7	0/6	1/17	1/30	*MT-CO3*	T –41– T	synonymous	yes
C9365T	0/7	1/6	0/17	1/30	*MT-CO3*	T –53– T	synonymous	yes
G9380A	0/7	0/6	1/17	1/30	*MT-CO3*	W –58– W	synonymous	yes
**G9477A**	**1/7**	**1/6**	**1/17**	**3/30**	***MT-CO3***	**V –91– I**	**missense**	**yes**
T9698C	0/7	3/6	0/17	3/30	*MT-CO3*	L –164– L	synonymous	yes
**Sum of detected changes in *MT-CO1, MT-CO2, MT-CO3***	8	15	17	39				
G8959A	***0/7***	***1/6 (4)***	***0/17***	***1/30***	***MT-ATP6***	***E –145– K***	***missense***	***no***
T8614C	1/7	0/6	0/17	1/30	*MT-ATP6*	L –30– L	synonymous	yes
T8715C	0/7	0/6	1/17	1/30	*MT-ATP6*	T –63– T	synonymous	yes
G8697A	1/7	1/6	0/17	2/30	*MT-ATP6*	M –57– M	synonymous	yes
C8818T	1/7	0/6	1/17	2/30	*MT-ATP6*	L –98– L	synonymous	yes
**A8860G**	**7/7**	**6/6**	**17/17**	**30/30**	***MT-ATP6***	**T –112– A**	**missense**	**yes**
G8994A	1/7	0/6	0/17	1/30	*MT-ATP6*	L –156– L	synonymous	yes
**G9055A**	**1/7**	**2/6**	**0/17**	**3/30**	***MT-ATP6***	**A –177– T**	**missense**	**yes**
A9093G	1/7	1/6	0/17	2/30	*MT-ATP6*	T –189– T	synonymous	yes

G9123A	0/7	0/6	1/17	1/30	*MT-ATP6*	L $\xrightarrow{199}$ L	synonymous	yes
**Sum of detected changes in *MT-ATP6***	13	10	20	43				
