# Supplementary material for: Mitochondrial DNA Changes in Genes of Respiratory Complexes III, IV and V Could Be Related to Brain Tumours in Humans
Source: Int J Mol Sci. 2022 Oct 12;23(20):12131. doi: 10.3390/ijms232012131 (PMC9603055; doi:10.3390/ijms232012131)
Supplement: Supplementary file 1 [file ijms-23-12131-s001.zip › Table S2.pdf]

Table S2. Biochemical values of the cytochrome b, subunits of complex IV and the ATP6 subunit depending on the presence of amino acid residues changes in a given protein position. Mutations are marked in bold italic**.**

Change in protein position	Theoretical isoelectric point	Aliphatic index	Instability index	Grand average of hydropathicity (GRAVY)	The helix percentage	Position in protein structure
**Cyt *b***						
I7T<br><br>(T14766C)	7.83	119.92	41.19 protein unstable	0.644	H1=0.00 alpha helix	transmembrane section
H16R<br><br>(A14793G)	8.42	118.89	42.18 protein unstable	0.627	H2 = 0.15 alpha helix	transmembrane section
F18L<br><br>(T14798C)	7.83	119.92	41.02 protein unstable	0.633	H2 = 0.11 alpha helix	transmembrane section
T158A<br><br>(A15218G)	7.83	119.16	40.80 protein unstable	0.637	H11 = 0.14 alpha helix	transmembrane section
T194A<br><br>(A15326G)	7.83	119.16	41.02 protein unstable	0.637	H12 = 4.59 alpha helix	transmembrane section, low complexity area
L236I<br><br>(C15452A)	7.83	118.89	41.53 protein unstable	0.633	H14 = 13.36 alpha helix	transmembrane section, low complexity area
S238F<br><br>(C15459T)	7.83	118.89	41.02 protein unstable	0.640	H14 = 32.29 alpha helix	transmembrane section, low complexity area
I304V<br>(A15656G)	7.83	118.63	41.61 protein unstable	0.630	H17 = 0.00 310 helix	transmembrane section, low complexity area
I306T<br><br>***(T15663C)***	***7.83***	***117.87***	***40.52 protein unstable***	***0.617***	***H17 = 0,00 310 helix***	***transmembrane section, low complexity area***
I338V<br><br>(A15758G)	7.83	118.63	40.58 protein unstable	0.630	H19 = 2.50 alpha helix	transmembrane section
**Normal protein**	7.83	118.89	41.02 protein unstable	0.693	H1 = 0.00<br>H2 = 0.09<br>H11 = 0.11<br>H12 = 2.00<br>H14 = 14.50<br>H17 = 0.00<br>H19 = 2.51	
**CO3**						
V91I<br>(G9477A)	6.78	92.30	23.70 protein stable	0.378	H3 = 0.25 alpha helix	transmembrane section, low complexity area
**Normal protein**	6.78	91.92	22.96 protein stable	0.377	H3 = 0.25	
**CO1**						
STP514K<br><br>(G7444A)	6.29	103.97	28.93 protein stable	0.674	-	mitochondrial matrix
G391A<br>(G7075C)	6.19	104.37	28.97 protein stable	0.687	H19 = 0.47 alpha helix	transmembrane section
**Normal protein**	6.19	104.17	28.97 protein stable	0.682	H19 = 0.25	
**ATP6**						

T112A (A8860G)	10.09	143.19	35.26 protein stable	0.912	H9 = 0.12 alpha helix	transmembrane section
E145K (G8959A)	***10.35***	***142.75***	***32.97 protein stable***	***0.900***	***beta roll***	***transmembrane section***
A177T (G9055A)	10.09	142.31	35.26 protein stable	0.890	H10 = 1.05 alpha helix	transmembrane section
**Normal protein**	10.09	142.75	35.26 protein stable	0.901	H9 = 0.07<br>H10 = 1.09	
