# Supplementary material for: Mitochondrial DNA Changes in Genes of Respiratory Complexes III, IV and V Could Be Related to Brain Tumours in Humans
Source: Int J Mol Sci. 2022 Oct 12;23(20):12131. doi: 10.3390/ijms232012131 (PMC9603055; doi:10.3390/ijms232012131)
Supplement: Supplementary file 1 [file ijms-23-12131-s001.zip › Table S3.pdf]

Table S3.The incidence of amino acid residue changes depending on protein position, assessed using PSSM viewer for cytochrome b, subunits of complex IV and the ATP6 subunit. Bold type indicates an amino acid that appears in the reference sequence in mtDB - Human Mitochondrial Genome Database (http://www.mtdb.igp.uu.se/; Uppsala, Sweden). Normal font indicates the amino acid in the test material. Mutations are marked in italic.

Fr (raw frequency/ unweighted frequency) – initial frequency - these are the initial frequency bars showing the real (unweighted) residual frequencies at each position of the seed alignment in conserved domains (CD). The characters in the gaps are treated as normal amino acids in these calculations; Fw (weighted frequency) - the frequency weighted column shows the calculated frequencies using the procedure Henikoff, JG. J Mol Biol. 1994; 243:574-578, modified as described on page 3395 of Altschul SF, et al. Nucleic Acids Res. 1997; 25: 3389-3402. Briefly, sequences similar in seed alignment have less weight in calculating the frequency of the residues as these sequences provide redundant information.

**Cyt *b* (MTH00100)**			
The rest of the amino acid	F_r	F_w	PSSM score
I7T			
T	0,55	0,51	5
**I**	**0,02**	**0,02**	**-2**
H16R			
R	0,01	0,02	0
**H**	**0,39**	**0,45**	**8**
F18L			
L	0,16	0,21	2
**F**	**0,84**	**0,79**	**8**
T158A			
A	0,01	0,02	-1
**T**	**0,90**	**0.88**	**6**
T194A			
A	0,19	0,15	0
**T**	**0,10**	**0,17**	2
L236I			
I	0,51	0,45	4
**L**	**0,32**	**0,41**	**4**
S238F			
F	0,10	0,07	0
**S**	**0,03**	**0,03**	**0**
I304V			
V	0,07	0,04	2
**I**	**0,63**	**0,66**	6
I306T			
T	*0.02*	*T*	*0.02*
I	***0.05***	***I***	***0.05***
I338V			
V	0,00	0,01	2
**I**	**1,00**	**0,99**	**7**
**CO1 (cd01663)**			
The rest of the amino acid	F_r	F_w	PSSM scores
G391A			
A	0.59	0.52	5
**G**	**0.14**	**0.14**	**2**
**CO3 (cd01665)**			
The rest of the amino acid	F_r	F_w	PSSM scores
V91I			
I	0.17	0.15	3
**V**	**0.55**	**0.52**	**6**
**ATP 6 (MTH00101)**			
The rest of the amino acid	F_r	F_w	PSSM scores
T112A			
A	0.32	0.24	2
**T**	**0.68**	**0.76**	**6**
E145K			
K	**-**	**-**	**-**
E	***1.00***	***1.00***	*7*

A177T			
T	0.07	0.11	1
**A**	**0.92**	**0.87**	**6**
