# Supplementary material for: Mitochondrial DNA Changes in Genes of Respiratory Complexes III, IV and V Could Be Related to Brain Tumours in Humans
Source: Int J Mol Sci. 2022 Oct 12;23(20):12131. doi: 10.3390/ijms232012131 (PMC9603055; doi:10.3390/ijms232012131)
Supplement: Supplementary file 1 [file ijms-23-12131-s001.zip › Table S4.pdf]

Table S4. Assessment of the conservativeness of amino acid residue changes in cytochrome b, in the subunit of complex IV and ATP6 subunit and of their effects on protein function using SIFT Sequence and ConSurf Database. Mutations are marked in italic. Scoring according to SIFT Sequence: change tolerated > 0.05, change affects protein function ⩽ 0.05. Conservativeness on a scale of 1 - 9: 1 - 3 variable region, 4 - 6 region of medium conservativeness, 7 - 9 region highly conserved. Conservativeness by normalized score: variable region < 0, region moderately conserved 0 - 0.5, highly conserved region > 0.5.

Change in protein position	Nucleotide sequence change	The frequency of occurrence of the reference sequence from mtDB – Human Mitochondrial Genome Database	The frequency of occurrence of the change sequence from mtDB – Human Mitochondrial Genome Database	Score value based on evaluation of the effect on protein function in SIFT Sequence (harmfulness of the change)	Conservativeness - normalized scores	Conservativeness - on a scale of 1 - 9
**Cyt *b***						
I7T	T14766C	2094	610	1.00 (tolerated)	1.193	2
H16R	A14793G	2665	39	0.01 (may affects the functioning of the protein)	1.516	1
F18L	T14798C	2486	218	4.22 (tolerated)	0.608	3
T158A	A15218G	2665	38	0.02 (may affects the functioning of the protein)	2.537	1
T194A	A15326G	17	2687	0.36 (tolerated)	1.371	1
L236I	C15452A	2469	235	1.00 (tolerated)	0.721	3
S238F	C15459T	2703	1	4.22 (tolerated)	1.178	2
I304V	A15656G	-	-	0.57 (tolerated)	0.155	5
I306T	*T15663C*	*2695*	*9*	*0.06 (tolerated)*	*-0.083*	*5*
I338V	A15758G	2675	29	0.01 (affects the functioning of the protein)	0.214	4
**CO1**						
G391A	G7075C	-	-	1.00 (tolerated)	-0.376	6
**CO3**						
V91I	G9477A	2606	98	0.19 (tolerated)	-0.298	6
**ATP6**						
T112A	A8860G	6	2698	0.20 (tolerated)	-0.463	6
E145K	*G8959A*	-	-	*0.00 (affects the functioning of the protein)*	*-1.475*	*9*
A177T	G9055A	2570	134	0.16 (tolerated)	-0.360	6
