# Supplementary material for: Mitochondrial DNA Changes in Genes of Respiratory Complexes III, IV and V Could Be Related to Brain Tumours in Humans
Source: Int J Mol Sci. 2022 Oct 12;23(20):12131. doi: 10.3390/ijms232012131 (PMC9603055; doi:10.3390/ijms232012131)
Supplement: Supplementary file 1 [file ijms-23-12131-s001.zip › Table S5.pdf]

Table S5. Assessment of the pathogenicity of amino acid residue changes in the cytochrome b, in the subunits of complex IV and in the ATP6 subunit using MitImpact3D (APOGEE predictor). APOGEE scoring: neutral change ⩽ 0.5, pathogenic change > 0.5. Mutations are marked in italic. Pathogenic change are marked in bold.

Change in protein position	Nucleotide sequence change	Pathogenicity (scoring according to APOGEE)
**Cyt *b***		
I7T	T14766C	Neutral (0.5)
H16R	A14793G	Neutral (0.25)
F18L	T14798C	Neutral (0.34)
T158A	A15218G	Neutral (0.26)
T194A	A15326G	Neutral (0.4)
L236I	C15452A	Neutral (0.36)
S238F	C15459T	Neutral (0.33)
I306T	*T15663C*	*Neutral (0.39)*
I304V	A15656G	Neutral (0.4)
**I338V**	**A15758G**	**Pathogenic (0.61)**
**CO1**		
G391A	G7075C	Neutral (0.49)
**CO3**		
V91I	G9477A	Neutral (0.34)
**ATP6**		
T112A	A8860G	Neutral (0.27)
E145K	*G8959A*	*Neutral (0.46)*
A177T	G9055A	Neutral (0.2)
