# Supplementary material for: Mitochondrial DNA Changes in Genes of Respiratory Complexes III, IV and V Could Be Related to Brain Tumours in Humans
Source: Int J Mol Sci. 2022 Oct 12;23(20):12131. doi: 10.3390/ijms232012131 (PMC9603055; doi:10.3390/ijms232012131)
Supplement: Supplementary file 1 [file ijms-23-12131-s001.zip › Table S6.pdf]

Table S6. Clinical characteristics of patients. *IDH* – gene of isocitrate dehydrogenase.

**Patient number**	**Age**	**Histopathological diagnosis**	**Mutation *IDH1***	**Mutation *IDH2***	**The size of the tumor (mm)**	**The location of the tumor**
**Grade IV**						
1	51	Glioblastoma multiforme with poorly differentiated component	-	-	34x31x26	right parietal lobe
2	57	Glioblastoma multiforme	-	-	44x36x30	right temporal lobe
3	49	Glioblastoma multiforme	R132H (c.395 G>A)	R172K (c. 515 G>A)	24x34x35	right frontal lobe
4	51	Glioblastoma multiforme	-	-	33x30x26	the right frontal-eclipse area
5	68	Glioblastoma multiforme	-	-	45x34x36	right occipital lobe
6	52	Glioblastoma multiforme	-	-	70x51x60	left temporal lobe
7	66	Glioblastoma multiforme	-	-	52x44x47	right temple-left-frontal-parietal area
8	58	Glioblastoma multiforme	-	-	60x45x50	right temporal lobe
9	43	Glioblastoma multiforme with a giant cell component	-	-	62x57x46	left frontal lobe
10	66	Glioblastoma multiforme	-	-	51x45x34	right occipital lobe
11	46	Glioblastoma multiforme	-	-	32x30x31	left frontal lobe
12	56	Glioblastoma multiforme	-	-	36x21x19	right temporal lobe
13	69	Glioblastoma multiforme	-	-	80x70x70	right temporal lobe
14	66	Glioblatoma multiforme with a primitive neuronal component	-	-	60x40x55	left parietal lobe
15	76	Glioblastoma multiforme	-	-	48x40x38	left parietal lobe
16	71	Glioblatoma multiforme with a primitive neuronal component	-	-	46x47x36	left temporal lobe
17	48	Glioblastoma multiforme	-	-	34x30x25	left parietal-occipital area
**Grade III**						
18	40	Pilocytic astrocytoma with anaplasia	-	-	33x29x25	right temporal lobe
19	39	Anaplastic astrocytoma	-	-	23x20x28	right temporal lobe
20	48	Anaplastic astrocytoma	R132H (c. 395 G>A)	-	54x51x52	the right occipital – parietal area and the body of the corpus callosum
21	68	Anaplastic astrocytoma	-	-	45x39x35	the right frontal-eclipse area
22	34	Anaplastic astrocytoma	-	-	61x56x68	right frontal lobe
23	37	Anaplastic oligodendroglioma	-	-	74x64x50	right temporal-frontal-parietal area
**Grade II**						
24	22	Astrocytoma gemistociticum	-	-	28x25x16	right temporal lobe
25	32	Astrocytoma diffusum	R132H (c. 395 G>A)	-	21x20x14	left temporo-fronto-parietal area
26	43	Astrocytoma diffusum	-	-	50x40x35	right frontal lobe
27	41	Oligodendroglioma	-	-	20x15x10	right frontal lobe and frontal – parietal area
28	52	Astrocytoma diffusum	-	R172S (c.516 G>T)	60x70x60	right frontal lobe
29	29	Oligodendroglioma	-	-	28x20x15	right frontal lobe
30	28	Oligodendroglioma	-	-	50x40x35	right parietal-occipital area
